# The impact of ‘on-pack’ pictorial health warning labels and calorie information labels on drink choice: A laboratory experiment

**DOI:** 10.1016/j.appet.2019.104484

**Published:** 2020-02-01

**Authors:** Eleni Mantzari, Rachel Pechey, Saphsa Codling, Olivia Sexton, Gareth J. Hollands, Theresa M. Marteau

**Affiliations:** Behaviour and Health Research Unit, University of Cambridge, Forvie Site, Robinson Way, CB2 0SR, Cambridge, UK

**Keywords:** Health warning labels, Calorie labels, Graphic warnings, Pictorial labels, Energy information, Sugar-sweetened beverages, SSBs, On-pack, SSBs, Sugar-sweetened beverages, SEP, Socio-economic position

## Abstract

Sugar-sweetened beverages (SSBs) are one of the largest added sugar sources to diets in the UK and USA. Health warning labels reduce hypothetical selection of SSBs in online studies but uncertainty surrounds their impact on selection of drinks for consumption. Calorie information labels are also promising but their impact on SSB selection is unclear. This laboratory study assessed the impact on SSB selection of ‘on-pack’ labels placed directly on physical products: *i.*a pictorial health warning label depicting an adverse health consequence of excess sugar consumption; and *ii.*calorie information labels. Potential moderation of any effects by socio-economic position (SEP) was also examined. Participants - 401 adults, resident in England, approximately half of whom were of lower SEP and half of higher SEP, were asked to select a drink from a range of two non-SSBs and four SSBs (subsequent to completing a separate study assessing the effects of food availability on snack selection). The drinks included ‘on-pack’ labels according to randomisation: Group 1: pictorial health warning label on SSBs; Group 2: calorie information label on all drinks; Group 3: no additional label. The primary outcome was the proportion of participants selecting an SSB. Compared to not having additional labels (39%), neither the pictorial health warning label (40%) nor calorie information labels (43%) affected the proportion of participants selecting an SSB. Lower SEP participants (45%) were more likely to select an SSB compared to those of higher SEP (35%), but SEP did not moderate the impact of labels on drink selection. In conclusion, pictorial health warning labels may be less effective in reducing SSB selection in lab-based compared with online settings, or depending on label design and placement. Findings suggest that effects might be absent when choosing from real products with actual ‘on-pack’ labels, positioned in a ‘realistic’ manner. Field studies are needed to further assess the impact of ‘on-pack’ SSB warning labels in real-world settings to rule out the possible contribution of study design factors.

## 1 Background

Sugar-sweetened beverages (SSBs) are one of the largest sources of added sugar to diets in the UK and USA (Bailey, Fulgoni, Cowan, & Gaine, 2018; Public Health England, 2018). Their consumption is linked to the development of adverse health conditions, including obesity, diabetes and dental decay (Bachman, Baranowski, & Nicklas, 2006; Batt et al., 2014; Bomback et al., 2010; Cohen, Curhan, & Forman, 2012; Fung et al., 2009; Larsson, Åkesson, & Wolk, 2014; Malik et al., 2006, 2010a, 2010b; Mishra & Mishra, 2011; Schernhammer et al., 2005; Te Morenga, Mallard, & Mann, 2013; Vartanian, Schwartz, & Brownell, 2007). Health warning labels could help reduce SSB purchasing and consumption, having been shown to be effective in improving a range of outcomes when used on tobacco products, including cessation-related behaviours (Borland, 1997; Borland & Hill, 1997; Hammond et al., 2003, 2004, 2006; Ngo, Cheng, Shang, Huang, & Chaloupka, 2018).

Although the use of health warning labels on sugary drinks has been considered by local and national policy-makers in the USA and UK (HM Government, 2016; Samuel, 2016), this is based on limited evidence. Most evidence to date consists of online studies involving hypothetical selection, with few known studies assessing actual drink selection or purchasing with health warning labels placed directly on SSB products. Nonetheless, these online studies highlight the potential of using health warning labels on beverages e.g. text-based health warning labels have been shown to be effective in improving understanding of the health harms associated with SSB overconsumption and may reduce the selection of such drinks (Bollard, Maubach, Walker, & Mhurchu, 2016; Gray, Karnon, & Blackwell, 2011; Roberto, Wong, Musicus, & Hammond, 2016; VanEpps & Roberto, 2016). The results of a recent laboratory study, conducted in a realistic purchasing context, also support the effectiveness of text-based labels, which were found to reduce the calories purchased from SSBs (Grummon, Taillie, Hall, Ranney, & Brewer, 2019). Pictorial health warning labels appear superior to text-based labels, having been shown to be more effective at reducing intentions to purchase SSBs and preferences for SSBs (Adams, Hart, Gilmer, Lloyd-Richardson, & Burton, 2014; Bollard et al., 2016). The results of the only existing field study to date confirm the superiority of pictorial health warning labels, which were found to be more effective than text-based labels in reducing sugary drink purchases (Donnelly, Zatz, Svirsky, & John, 2018). It is worth noting, however, that in this study the labels were placed on shelves immediately below the sugary drinks rather than on the products themselves.

Not all pictorial warnings, however, might be equally effective. Based on evidence from the use of warning labels on tobacco products, the most effective labels are those that use images that elicit a strong negative emotional response (Cho et al., 2017; Hammond et al., 2003, 2004, 2006; Nonnemaker, Choiniere, Farrelly, Kamyab, & Davis, 2014). Consistent with this, health warning labels that include images illustrating the negative health consequences of excess sugar consumption are more effective in reducing SSB selection in hypothetical choice scenarios (Billich et al., 2018; Mantzari, Vasiljevic, Turney, Pilling, & Marteau, 2018), even when compared to other types of pictorial labels, such as those that include images illustrating drink sugar content (Mantzari et al., 2018). This accords with evidence suggesting that images of negative health consequences of consumption make attitudes towards unhealthy foods less favourable and that these attitudes mediate effects on selection (Hollands & Marteau, 2016). In situations in which actual drink choices are made, it remains to be determined whether ‘on pack’ pictorial health warning labels applied directly to the products themselves are more effective than other health warning labels in reducing actual, rather than hypothetical SSB selection.

The use of labels conveying calorie information has also been recommended as a way to facilitate healthier choices (WHO, 2014), having the potential to change people's choices at the point of selection or consumption when placed on menus or adjacent to products (Crockett et al., 2018). With regard to the impact of calorie information labels on SSB purchasing and consumption, findings are mixed. Two field studies have shown that they can reduce SSB selection when information is provided in the form of physical activity equivalents, such as minutes of running required to burn off the energy contained in a bottle of soda (Bleich et al., 2012, 2014). When given in the form of calories per bottle, two online studies and a recent field study show that such labels have no effect on selection (Mantzari et al., 2018; Roberto et al., 2016) or purchasing (Donnelly et al., 2018). These studies, however, either assessed hypothetical choices (Mantzari et al., 2018; Roberto et al., 2016) or involved placing the labels on shelves rather than directly on the drinks (Donnelly et al., 2018). Further research, therefore, is needed, conducted in real-world settings, to assess the impact of directly labelling single drink products.

The current laboratory study aimed to assess the impact of ‘on-pack’ health warning labels and calorie information labels on actual, rather than hypothetical selection of SSBs. The primary aim was to assess the impact on SSB selection of: *i.* an ‘on-pack’ pictorial health warning label depicting an adverse health consequence of excess sugar consumption; *ii.* ‘on-pack’ calorie information labels. As SSB consumption is socially patterned, thereby contributing to observed inequalities in health outcomes (Bolt-Evensen, Vik, Stea, Klepp, & Bere, 2018; Han & Powell, 2013; Lobstein, 2014; Mazarello Paes et al., 2015; Pabayo, Spence, Cutumisu, Casey, & Storey, 2012), the study also aimed to assess whether any effect on SSB selection of ‘on-pack’ health warning labels or calorie information labels was moderated by socio-economic position in a way that might reduce health inequalities (Sarink et al., 2016).

## 2 Methods

### 2.1 Design

Randomised controlled between-subjects study in a laboratory setting, in which participants were randomly allocated to one of three groups (see Interventions).

### 2.2 Participants

Participants were 401 adults, resident in England, taking part in a separate study assessing the impact of food availability on food selection (study registration available here https://osf.io/zn567/). They were recruited by a market research company (Roots Research; https://rootsresearch.co.uk) and purposefully sampled to ensure an approximately even split between individuals of higher and of lower socioeconomic position (SEP), as defined by highest educational qualification: higher SEP was defined as having a degree or having completed higher education; lower SEP was defined as having completed up to GCSEs or equivalent. The characteristics of participants across groups are shown in Table 1.

**Table 1:** Characteristics of study participants (n (%)).

	Group 1: Pictorial health warning label (n = 136)	Group 2: Calorie information label (n = 131)	Group 3: No additional label (n = 134)	Total (n = 401)
Age (sd)	39.3 (13.4)	40.3 (13.7)	40.5 (14.7)	40.0 (13.9)
Gender				
Female	79 (58%)	70 (53%)	75 (56%)	224 (56%)
Male	57 (42%)	61 (47%)	59 (44%)	177 (44%)
Ethnicity				
White	114 (84%)	114 (87%)	117 (87%)	345 (86%)
Black	3 (2%)	4 (3%)	1 (1%)	8 (2%)
Asian	12 (9%)	7 (5%)	13 (10%)	32 (8%)
Mixed	5 (4%)	3 (2%)	2 (1.5%)	10 (2.5%)
Other/Prefer not to say	1 (1%)	3 (2%)	1 (1%)	5 (1%)
Income				
Under £15,500	24 (17.6%)	14 (10.7%)	23 (17.2%)	61 (15%)
£15,500-£24,999	21 (15.4%)	19 (14.5%)	18 (13.4%)	58 (14.5%)
£25,000-£39,999	31 (22.8%)	34 (26%)	30 (22.4%)	95 (24%)
£40,000- £49,999	17 (12.5%)	19 (14.5%)	18 (13.4%)	54 (13.5%)
£50,000-£74,999	14 (10.3%)	21 (16%)	19 (14.2%)	54 (13.5%)
Above £75,000	17 (12.5%)	14 (10.7%)	13 (9.7%)	44 (11%)
Don't know/prefer not to say	11 (8.1%)	10 (7.6%)	13 (9.7%)	34 (8.5%)
Socioeconomic position				
Higher	65 (48%)	61 (47%)	65 (48.5%)	191 (48%)
Lower	71 (52%)	70 (53%)	69 (51.5%)	210 (52%)
Healthiness of snack chosen				
Healthier	60 (44%)	59 (45%)	58 (43%)	177 (44%)
Less healthy	76 (56%)	72 (55%)	76 (57%)	224 (56%)

### 2.3 Sample size calculations

The study sample size was opportunistic. Previous research has found a reduction in the proportion of participants selecting an SSB of 16.8% (49.2% vs 32.4%) with use of a pictorial health warning label (illustrating rotting teeth) compared to not using any label (Mantzari et al., 2018). A sample of 401 participants (approximately 133 in each group) provided 80% power to detect this difference at the 5% statistical significance level.

### 2.4 Interventions

Participants were randomised to one of three groups and invited to select a beverage from a range of six, comprising four SSBs and two non-SSBs that varied in the addition of an ‘on-pack’ label (*i.* placed on the products themselves) to the drinks offered: Group 1: pictorial health warning label on SSBs only; Group 2: calorie information label on all drinks; Group 3: no additional label. Interventions involving health warning or calorie labels applied to product packaging are categorised as an Information x Product intervention within the TIPPME intervention Typology (Hollands et al., 2017). Randomisation was stratified according to SEP to ensure a balance between groups and was performed by a statistician independent of the research team.

#### 2.4.1 Pictorial health warning label

**Fig. 1:**
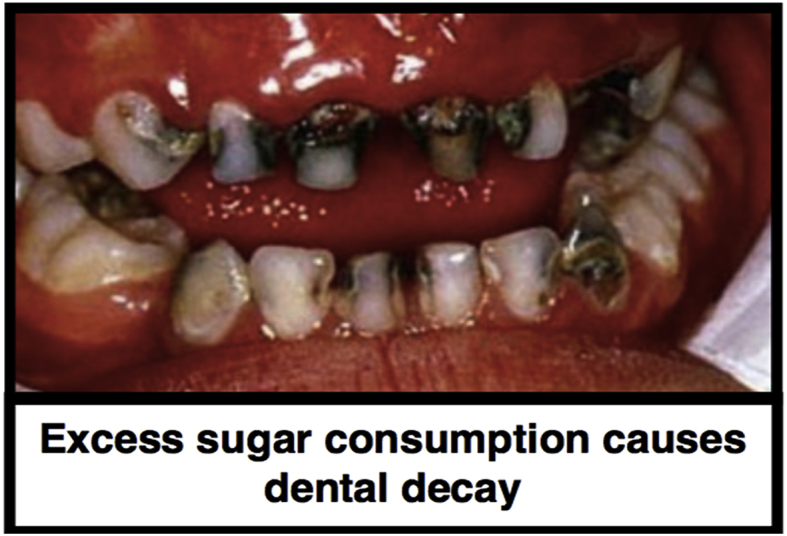
Pictorial health warning label.

This comprised an image of rotting teeth alongside the caption “Excess sugar intake causes dental decay” (Fig. 1) affixed on the left of the logo of each SSB presented to participants. The height of the labels was designed to cover the height of the manufacturers’ labels and was between 4.5 cm and 5 cm, depending on the drink. The width was 4.7 cm. This label was chosen based on the results of an online study, which showed it to be the most effective in reducing SSB selection by parents choosing a drink for their children in a hypothetical choice task (Mantzari et al., 2018).

#### 2.4.2 Calorie information label

**Fig. 2:**
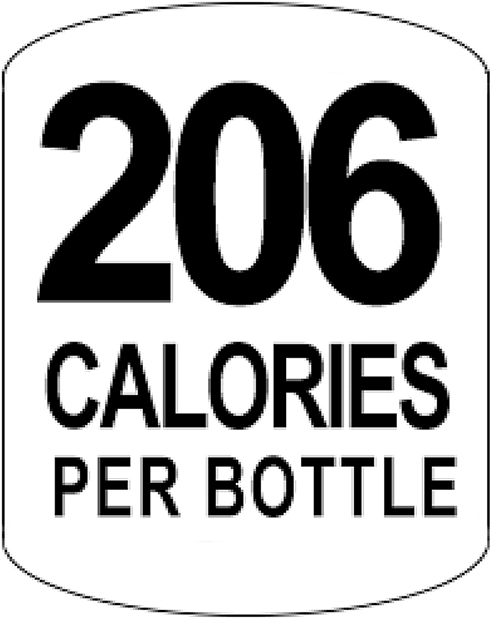
Example of calorie information label. The number of calories differed for each drink.

This comprised the number of calories per bottle contained in the drink. The number was shown using numerals in black font against a white background and affixed on the left of the logo of each drink (both SSBs and non-SSBs) presented to participants (See Fig. 2 for an example of the calorie information label. The number of calories differed for each drink and ranged from 210 to 0). The height of the labels was designed to cover the height of the manufacturers’ labels and was between 3.2 cm and 5 cm, depending on the drink. The width was 3 cm.

Both the pictorial health warning label and the calorie information labels were printed as stickers on 70-μm MD-5 High Performance Polymeric Calendered Gloss Vinyl.

#### 2.4.3 No additional label

No additional label or information was added to any of the six drinks offered.

### 2.5 Measures

#### 2.5.1 Primary outcome

Proportion of participants choosing an SSB.

#### 2.5.2 Predictors

• Socio-economic position (SEP), assessed by highest educational qualification
• Healthiness of snack chosen in a preceding study assessing the impact of food availability on food selection (categorised as either healthier i.e. containing 100 calories or less or less healthy a i.e. containing more than 200 calories) (See Procedure).

#### 2.5.3 Other measures

Demographic characteristics:

• Age
• Gender
• Ethnicity
• Income

### 2.6 Procedure

Participants were invited to a generic function room hired in a church in central Cambridge, where they were provided with information about the study and gave written ‘informed’ consent for participation. Participants were not told the true purpose of the study, as it was considered that revealing the true aims of the study might influence any drink choices. Instead they were told that they were participating in a study to investigate the effect of snacking on cognitive performance and that drinks would be offered after consumption of a snack in order to ‘wash it down’.

Participants completed the current study following a separate study assessing the effects of the availability of healthier and less healthy food items on snack selection. During that study, participants were randomly assigned to one of three conditions, differing in the number of healthier and less healthy snacks to choose from: a control condition in which participants were presented with equal numbers of healthier and less healthy snack options (2 of each); an intervention condition in which participants were presented with an increased number of healthier snack options (6 healthier, 2 less healthy); or an intervention condition, in which participants were presented with an increased number of less healthy snack options (2 healthier, 6 less healthy).

**Fig. 3:**
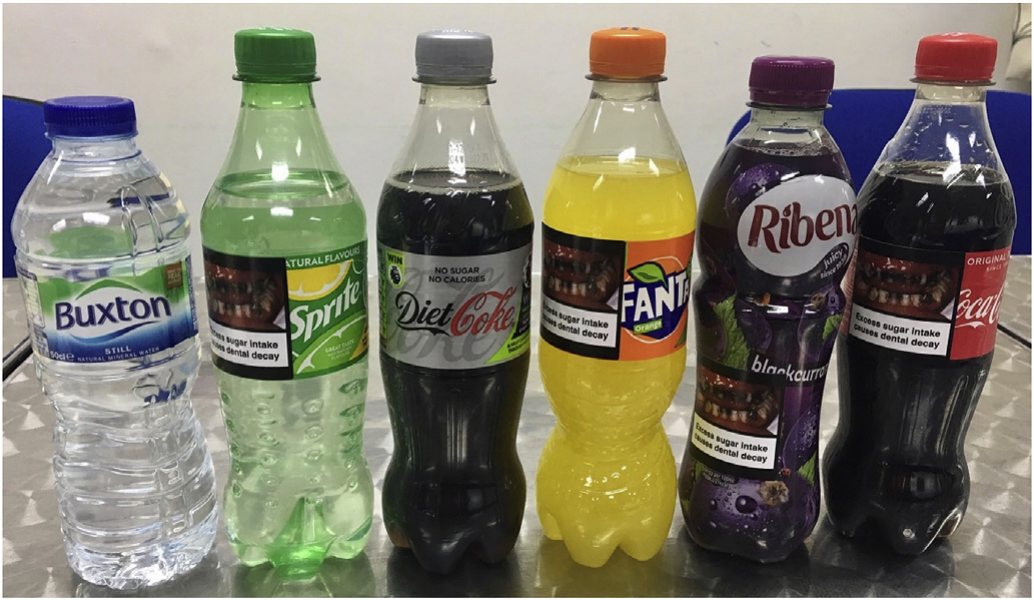
Pictorial health warning labels placed on SSBs.

**Fig. 4:**
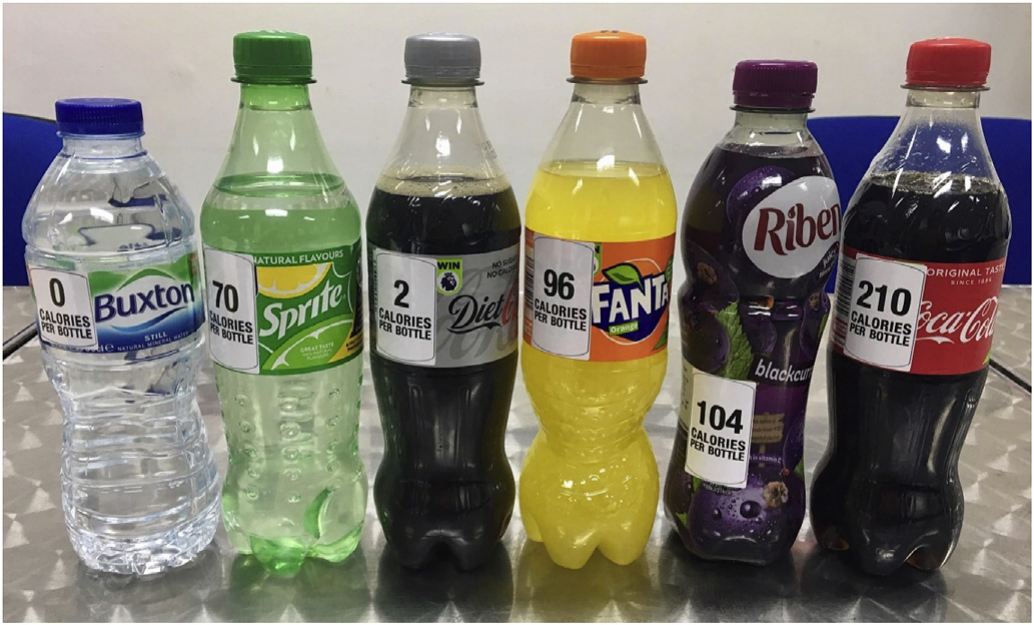
Calorie information label on SSBs and non-SSBs.

Following completion of that study and selection of their snack, participants were presented with a range of six drinks (4 SSBs: Coca Cola, Fanta, Sprite, Ribena, and 2 non-SSBs: Still Water (Buxton Natural Mineral Water) and Diet Coke) and asked to select one for immediate consumption. They were allowed not to select a drink if they preferred. They were also allowed to drink as much of their chosen beverage as they wanted or take it with them to consume at a later time. Depending on participants' allocated group, the drinks had (i) a pictorial health warning label on the four SSBs; (ii) calorie information labels on all drinks; or (iii) no additional labels. The drinks were placed on a table in a row, hidden from participants’ view by a box until it was time to choose a drink. Drinks were positioned in such a way as to allow any affixed labels to be visible when the drinks were revealed. The order in which the drinks were presented was kept constant for all participants (Water, Sprite, Diet Coke, Fanta, Ribena, Coca Cola). See Fig. 3, Fig. 4 for an illustration of how the labels were placed on the bottles.

After making their drink choice, participants were fully debriefed on the aims of the study and received between £30-£40 in a combination of ‘cash and Love2Shop vouchers’, or in ‘cash only’ to reimburse them for time spent taking part in both studies. The reimbursement amount increased from £30 to £40 towards the end of the study due to the quota for number of low SEP participants not being met.

### 2.7 Statistical analyses

Descriptive proportions of SSB selection with normal approximation 95%CI were calculated. Logistic regression analysis was performed to assess the odds of selecting an SSB in each group while adjusting for the healthiness (healthier vs less healthy) of the snack selected and consumed in the preceding study. To assess the potential moderating effect of SES on the effect of labels on SSB selection, the interaction between SEP and label group was added to the logistic regression model.

## 3 Results

There were no statistically significant differences between randomised groups in participant characteristics (Table 1). Eleven participants, six from Group 1 (pictorial health warning label), one from Group 2 (calorie information label) and four from Group 3 (no additional label), chose not to select any drink. These participants were included in the analysis. Excluding them from the analysis did not affect the results. Descriptive information regarding the proportions of participants choosing an SSB according to each group and socioeconomic position are shown in Table 2.

**Table 2:** Proportion of participants ((n) 95% CI) selecting an SSB by Group and Socio-Economic Position (SEP).

	Group 1: Pictorial health warning label	Group 2: Calorie information label	Group 3: No additional label	Total
	40% ((54/136) 31.5%–48.4%)	43% ((56/131) 34.2%–51.6%)	39% ((52/134) 30.6%–47.6%)	40% ((162/401) 35.5%–45.4%)
Higher SEP	38% ((25/65) 40.1%–70.0%)	38% ((23/61) 25.8%–51.0%)	29% ((19/65) 18.9%–42.0%)	35% ((67/191) 28.4%–42.3%)
Lower SEP	41% ((29/71) 29.5%–53.1%)	47% ((33/70) 35.2%–59.3%)	48% ((33/69) 35.7%–60.1%)	45% ((95/210) 38.4%–52.2%)

Neither the pictorial health warning label (40% vs 39%; OR = 1.500, 95%CI = 0.721, 3.121) nor the calorie information labels (43% vs 39%; OR = 1.461, 95%CI = 0.694, 3.078) significantly affected the odds of selecting an SSB compared to no additional label. Socio-economic position had a significant impact on SSB selection, with those of lower SEP being more likely to select an SSB compared to those of higher SEP (45% vs 35%; OR = 2.184, 95%CI = 1.068, 4.462). The pictorial health warning label and calorie information labels did not differentially affect SSB selection in those of lower or higher SEP, as indicated by the lack of a significant interaction effect (Table 3).

**Table 3:** ORs (95% CI) of choosing an SSB according to Group and Socio-Economic Position, adjusting for selected snack healthiness.

		B (SE)	OR	95% CI for OR	
				Lower	Upper
Group (ref: No additional label)	Calorie information label	0.379 (0.380)	1.461	0.694	3.078
	Pictorial health warning label	0.405 (0.374)	1.500	0.721	3.121
Socio-economic position (ref: Higher)	Lower	0.781 (0.365)	2.184*	1.068	4.462
Group by Socio-Economic Position	Calorie information label by Lower SEP	−0.396 (0.510)	0.673	0.248	1.830
	Pictorial health warning label by Lower SEP	−0.677 (0.507)	0.508	0.188	1.372
Snack Healthiness (ref: Healthier)	Less healthy	-.214 (0.208)	0.807	0.537	1.213

* Significant at the p < 0.05 level.

## 4 Discussion

In this laboratory-based study, the addition of an ‘on-pack’ pictorial health warning label or calorie information labels directly on SSB packaging did not reduce selection of SSBs. Although socio-economic position had a significant impact on SSB selection, with participants of lower SEP being more likely to select an SSB compared to those of higher SEP, the effects of the labels did not vary according to socio-economic position.

The findings of the current study are inconsistent with previous research demonstrating the effectiveness of health warning labels for reducing SSB selection (Adams et al., 2014; Billich et al., 2018; Bollard et al., 2016; Donnelly et al., 2018; Gray et al., 2011; Mantzari et al., 2018; Roberto et al., 2016; VanEpps & Roberto, 2016). There are various possible explanations for the differing findings. First, they may be the result of different study designs. Most prior research in the area consists of online studies in which hypothetical SSB selection has been assessed (Adams et al., 2014; Billich et al., 2018; Bollard et al., 2016; Gray et al., 2011; Mantzari et al., 2018; Roberto et al., 2016; VanEpps & Roberto, 2016). The findings of the current study raise the possibility that effects are reduced or diminished when assessing actual selection from an array of physical products. This possibility is not supported by the results of a recent field study and a recent laboratory study conducted in a realistic setting, in which health warning labels were found effective in reducing sugary drink purchases (Donnelly et al., 2018) and calories purchased from SSBs (Grummon et al., 2019), respectively. The labels in the field study were placed on shelves immediately below the sugary drinks rather than on the drinks themselves. In the laboratory study, they were placed in a prominent position on the front of bottles, in red colour, partially obstructing manufacturers' logos. These placement positions might have increased the visibility of the labels and the chances that they were noticed and thus deterred SSB selection Similarly, in the aforementioned online studies, visibility of the labels, which were digitally placed on images of SSB bottles, was ensured by including zoomed in images of the labels. The findings of the present study, therefore, might reflect the possibility that the labels were less visible than in previous studies, a possibility given that they were placed on the side of bottles, ensuring that they were in keeping with existing manufacturers' labels (i.e. as if they were part of the existing labels) and did not cover any of the branding and logos. This placement position – i.e. being part of the existing label and not obstructing logos and branding – was chosen on the basis of it being considered more realistic and feasible, if warning labels were to be implemented on SSBs. Although pictorial health warning labels generally attract more attention than text-based labels (Evans et al., 2015; Romer et al., 2017), noticeability of the pictorial warning labels in the current study might have been further hindered by their dark colour, which might have failed to attract attention in the same way as red-coloured text-based labels possibly did in the aforementioned laboratory study (Grummon et al., 2019). Using one bright colour, such as red, may not be feasible with pictorial labels. Using images, however, with high contrasts and bright colours could potentially increase visibility. A manipulation check was not included to assess label visibility. However, during debriefing, participants often commented that they hadn't noticed the labels. This provides support for the possibility that, depending on label format and placement, when placed on actual drinks in a ‘realistic’ manner, pictorial health warning labels might not always be effective in deterring actual SSB selection. Although front-of-pack labels might be more visible, their use on SSB bottles might not be feasible, given the placement of branding and logos and the potential legal restrictions to obstruct these. Further field studies assessing the impact of ‘on-pack’ health warning labels on selection of actual physical products are needed to inform their suggested use as an intervention to reduce the consumption of sugary drinks and foods. Such studies should also assess the design and placement of ‘on-pack’ health warning labels, taking into consideration potential restrictions from manufacturers and their need to be incorporated into existing labels, in the same way as is done on cigarette packets.

Another possibility for the inconsistent results is that the findings of the current study are due, at least in part, to the study sample. Previous studies assessing the impact of ‘on pack’ labels have targeted regular SSB consumers (Adams et al., 2014; Billich et al., 2018; Bollard et al., 2016; Gray et al., 2011; Grummon et al., 2019; Mantzari et al., 2018; Roberto et al., 2016; VanEpps & Roberto, 2016). The current study did not specifically recruit regular SSB drinks and SSB consumption frequency was not assessed. If the majority of participants were not regular SSB consumers, this might have affected the impact of the warning labels. This is possible given that 39% of those in the control group chose an SSB, compared to around 49% in a previous study with UK-based SSB consumers (Mantzari et al., 2018), and between 60% and 77% in studies with US-based SSB consumers (Grummon et al., 2019; Roberto et al., 2016; VanEpps & Roberto, 2016). Individuals not consuming SSBs on a regular basis might not be as concerned as regular SSB consumers about the health consequences associated with sugary drink consumption. As perceptions of personal risk can affect the effectiveness of risk information (Ferrer & Klein, 2015), it is possible that non-SSB drinkers may thus disregard or not take notice of the risk information displayed by warning labels. This might have been especially true in the current study, given the choice of health warning label i.e. an image of rotting teeth warning of the risk of dental decay. This label was selected based on the results a previous study assessing the impact of different image-based warning labels on SSB selection by parents choosing a drink for their child (Mantzari et al., 2018). Tooth decay, however, might not be of huge concern to adults selecting a drink for themselves, especially if they are not regular SSB drinkers. This is consistent with the results of the aforementioned field and realistic laboratory studies, which found effects using labels highlighting additional health consequences, including obesity and diabetes. The possibility that the lack of effects is due to the study not targeting regular SSB consumers is slightly mitigated by the fact that in the aforementioned field study, which was conducted in a hospital cafeteria, purchasing of SSBs was relatively low at baseline --21% of bottled drinks--, implying that most customers were non-SSB drinkers. Nonetheless, pictorial health warning labels significantly reduced purchasing of SSBs (Donnelly et al., 2018).

A further explanation for the inconsistent findings is that the results of the current study were affected, at least in part, by the artificial nature of the task. In contrast to the only field study and realistic laboratory study in the area, which assessed the impact of health warning labels on the purchasing of SSBs (Donnelly et al., 2018, Grummon et al., 2019), in the current study, a limited selection of drinks were given for free. This arguably reduced the ecological validity of the study and may have introduced social desirability effects, as suggested by the fact that most participants selected non-SSBs, even though the variety of non-SSBs offered was limited compared to the SSBs, in both quantity and flavour. Warning labels might have different effects on the behaviour of individuals choosing from a range of many beverages in a store and paying for a drink rather than receiving it for free, especially if they are thirsty – although having just consumed a snack, it could be argued that participants in the current study might also have been relatively thirsty. Alternatively, the results might reflect the fact that the drinks were presented immediately after participants had completed participation in a preceding study in which they had to complete a number of cognitively demanding tasks, including measures of response inhibition (the stop-signal task and the Stroop task) and an implicit attitudes task concerning food (the Implicit Association Task), as well as make a snack selection. Completion of these tasks might have affected their cognitive resources available to process the labels. According to dual processing models of decision making [e.g. (Strack & Deutsch, 2004)], low cognitive resource inhibits activation of the reflective system that generates behavioural decisions based on reasoning, judgment and knowledge about facts and values and increases activation of the impulsive system that elicits behaviour through associative links (Hinson, Jameson, & Whitney, 2002; Shiv & Fedorikhin, 1999). Under cognitive-load, therefore, people have less ability to process risk information and rely on heuristics to make satisfactory decisions with minimal effort (Friese, Hofmann, & Wänke, 2009; Hofmann, Gschwendner, Friese, Wiers, & Schmitt, 2008; Whitney, Rinehart, & Hinson, 2008). This could potentially affect the way labels are processed. For example, in a study in which cognitive load was deliberately manipulated, colour-coded nutritional labels were effective in promoting healthier food choices but only when cognitive resources were low (Werle). The opposite might be true for health warning labels, which may be more likely to require conscious engagement with the risk information presented, at least in terms of the text information that they contain (Hollands, Marteau, & Fletcher, 2016). It would be of interest in future research to assess the impact of health warning labels on SSB selection when available cognitive resource is high compared with low.

A final possible explanation for the lack of effect of the health warning labels in the current study is that the study was underpowered. Although it was estimated that the sample size would give sufficient power to detect an effect of the health warning label compared to not using additional labels, these estimates were based on research using a different study design (i.e. online study assessing hypothetical SSB selection) and different sample (i.e. parents of children, most of whom were of higher SEP) to the current study.

The findings presented here regarding the calorie information labels are consistent with previous online and field studies showing that such labels have no effect on selection (Mantzari et al., 2018; Roberto et al., 2016) or purchasing (Donnelly et al., 2018) of SSBs. Although calorie information labels have the potential to change people's choice at point of selection and consumption, when placed on menus or adjacent to products (Crockett et al., 2018), the results of the current study suggest that placing nutritional information on beverages, at least in the form of calories per bottle, may have little influence on selection. Calorie information provided in the form of physical activity equivalents such as minutes of running required to burn off the energy contained in a bottle of soda, might have greater potential (Bleich et al., 2012, 2014) and should be explored in further research. The results of the current study also confirm previous research showing that SSB consumption is socially patterned, with consumers of lower socio-economic position being more likely to select an SSB compared to consumers of higher socio-economic position (Bolt-Evensen et al., 2018; Han & Powell, 2013; Lobstein, 2014; Mazarello Paes et al., 2015; Pabayo et al., 2012). This highlights the continued need for interventions that will reduce SSB consumption in the population overall but especially amongst those who are more deprived. Although socio-economic position did not moderate the impact of the labels in the current study, caution is warranted against drawing conclusions regarding the effectiveness of health warning labels for reducing health inequalities, as the study was potentially underpowered to detect such moderation effects.

The research presented here contributes to the limited evidence on the use of warning labels on SSBs. The study is one of the very few to assess the impact of pictorial health warning labels and calorie information labels on SSBs on actual rather than hypothetical selection, and the only one to date to do so while using ‘on-pack’ pictorial health warning labels – as opposed to text-based labels – placed directly on beverages, in a manner that is arguably realistic and feasible for their winder implementation. Although the aforementioned design and power limitations restrict the conclusions that can be drawn, the findings provide valuable information regarding the potential of ‘on-pack’ warning and calorie labels to reduce SSB selection and therefore consumption. The study also raises important issues regarding the design and placement of labels and their implications on noticeability and thus effectiveness, which need to be addressed in future research. By also including an assessment of the moderating role of socio-economic position, the study provides information regarding the potential for warning labels to reduce the health inequalities associated with SSB consumption.

## 5 Conclusion

In conclusion, we found no evidence that pictorial health warning labels or calorie information labels reduced selection of SSBs in a lab-based setting involving actual selection of drinks. Although the contribution of study design factors cannot be excluded, the results suggest that depending on their format and placement, ‘on-pack’ pictorial health warning labels placed on SSBs in a ‘realistic’ manner may be less effective in reducing SSB selection when choosing from real products with actual ‘on-pack’ labels, than suggested by previous research. Calorie information labels also do not appear to influence choices. Field studies of ‘on-pack’ SSB warning labels placed directly on physical products are needed to further assess their potential impact on selection and consumption in real-world settings. Such studies should also explore the ideal label design and placement on SSBs, while also taking into consideration potential manufacturers' restrictions and the need to be incorporated into existing labels.

## Funding

This report is independent research commissioned and funded by the 10.13039/501100000272National Institute for Health Research Policy Research Programme (Policy Research Unit in Behaviour and Health [PR-UN-0409-10109]). The views expressed in this publication are those of the author(s) and not necessarily those of the NHS, the National Institute for Health Research, the Department of Health and Social Care or its arm's length bodies, and other Government Departments. RP is supported by a 10.13039/100010269Wellcome Trust Research Fellowship in Society and Ethics [106679/Z/14/Z].

## Ethics approval and consent to participate

Ethical approval was obtained by the University of Cambridge Psychology Department Research Ethics Committee (reference number Pre.2018.018). Written informed consent to participate in this study was obtained by all participates.

## Authors’ contributions

All authors collaborated in designing the study. RP, SC, and OS ran the study and collected the data. EM conducted the analysis. The manuscript was drafted by EM with substantial contributions from RP, SC, OS, GJH and TMM. All authors read and approved the final manuscript.

## Availability of data and materials

The data and materials are available upon request.

## Declaration of competing interest

The authors declare that they have no competing interests. This report is independent research commissioned and funded by the 10.13039/501100000272National Institute for Health Research Policy Research Programme. The views expressed in this publication are those of the author(s) and not necessarily those of the NHS, the National Institute for Health Research, the Department of Health and Social Care or its arm's length bodies, and other Government Departments.
